# Hydrogen-rich saline treatment modulates proteomic profiles to mitigate cataract development in a N-methyl-N-nitrosourea-induced rat model

**DOI:** 10.1007/s10792-025-03915-6

**Published:** 2026-01-20

**Authors:** Yingxin Qu, Xiaoqi Li, Qinghua Yang, Runpu Li, Ye Tao, Yifei Huang, Liqiang Wang

**Affiliations:** 1 Inner Mongolia Chaoju Eye Hospital, Hohhot, China; 2 Department of Ophthalmology, 971 Navy Hospital, Qingdao, China; 3 Senior Department of Ophthalmology, the Third Medical Center of PLA General Hospital,, Beijing, China; 4 Department of Ophthalmology, Eye Hospital of Henan, Zhengzhou, China; 5 Department of Ophthalmology, Chinese PLA General Hospital, Beijing, China

**Keywords:** Cataract, N-methyl-N-nitrosourea, Hydrogen, Oxidative stress, Tandem mass spectrometry, Proteomics

## Abstract

**Purpose:**

Cataracts are associated with oxidative stress-induced damage to lens proteins. This study aims to identify differentially expressed proteins (DEPs) associated with the protective effects of hydrogen-rich saline (HRS) against N-methyl-N-nitrosourea (MNU)-induced cataracts, utilizing the antioxidant properties of hydrogen.

**Methods:**

Sprague–Dawley rats were assigned to control, MNU-only, MNU + normal saline (NS), MNU + pirenoxine(PRX), and MNU + HRS groups. Cataracts were induced with MNU (postnatal day 15), and treatments (postnatal days 8–21) included intraperitoneal injections and eye drops. Cataract severity was assessed using slit-lamp examinations, Pentacam analysis, and spectrophotometry. Proteomic analysis of lens tissues from the MNU + HRS and MNU + NS groups employed tandem mass tag (TMT) labeling and mass spectrometry. DEPs were identified, grouped based on fold changes, and analyzed for Gene Ontology (GO), Kyoto Encyclopedia of Genes and Genomes (KEGG),, and domain enrichment. Parallel reaction monitoring (PRM) validated selected DEPs.

**Results:**

HRS reduced MNU-induced cataract incidence to 50% versus 100% in MNU-only and NS groups and preserved lens clarity comparable to normal controls. Proteomic analysis identified 90 upregulated and 303 downregulated proteins in the HRS-treated group versus the NS-treated group. DEPs were enriched in GO terms related to ion transport, homeostasis, and ATP hydrolysis, as well as KEGG pathways like oxidative phosphorylation and arginine biosynthesis. Domain enrichment showed links to ATPase activity and energy metabolism. DEPs were grouped into Q1–Q4, with Q1 showing enrichment in oxidative phosphorylation and metabolic pathways. PRM confirmed the downregulation of 14 stress-response and metabolic proteins in the HRS-treated group.

**Conclusion:**

HRS mitigates MNU-induced cataracts possibly by reducing oxidative stress and downregulating stress-response and metabolic proteins.

**Supplementary Information:**

The online version contains supplementary material available at 10.1007/s10792-025-03915-6.

## Introduction

Cataracts, the leading cause of blindness worldwide, are characterized by progressive lens opacification due to the aggregation of lens proteins, which compromises lens transparency and impairs vision [1]. Responsible for 50% of blindness in middle- and low-income countries, cataracts affect 95 million people globally, with prevalence rising due to aging populations [2]. While age-related cataracts are most common [3], oxidative stress plays a critical role in cataract formation by promoting protein aggregation, lipid peroxidation, and disruption of lens epithelial cell homeostasis [4]. Current treatment relies exclusively on surgical intervention to remove the opaque lens and replace it with an artificial intraocular lens. However, surgery addresses only the end-stage pathology without targeting the underlying mechanisms, such as oxidative damage, that drive cataract formation [5]. Furthermore, access to surgical treatment is limited in middle- and low-income countries due to high costs, shortages of trained surgeons, and inadequate infrastructure [6]. These limitations underscore the urgent need for pharmacological strategies, particularly antioxidant therapies, which could prevent or delay cataract progression and reduce the global burden of this condition [7].

Although antioxidant therapies such as vitamins C and E have shown potential in mitigating oxidative stress, their limited efficacy in reversing or delaying cataract progression highlights a critical gap in treatment strategies [8]. Among emerging candidates, molecular hydrogen, particularly in the form of hydrogen-rich saline (HRS) by dissolving hydrogen gas into saline, has demonstrated potent antioxidative, anti-inflammatory, and anti-apoptotic effects in preclinical studies [9]. The efficacy of HRS can be primarily attributed to its unique ability to scavenge specific reactive oxygen species (ROS) without perturbing cellular redox signaling or antioxidant defenses [10]. Moreover, HRS has been shown to modulate critical inflammatory pathways by altering the expression or activity of NF-κB and other cytokine signaling networks, thereby reducing pro-inflammatory responses [11]. The anti-apoptotic effects of HRS may be mediated through both the upregulation of cytoprotective genes and proteins like Bcl-2 and the inhibition of apoptotic pathways involving caspases [12]. Despite these promising attributes, the detailed mechanisms by which HRS influences cataract progression remain underexplored, necessitating further investigation into its potential to address oxidative stress-induced lens damage. Identifying differentially expressed proteins (DEPs) in response to HRS treatment may elucidate its mechanisms and the pathways involved in cataract prevention.

To evaluate the efficacy of HRS and provide a benchmark for comparison, we included pirenoxine (PRX) as a positive control in our experimental design. PRX is a clinically used ophthalmologic agent for cataract treatment and prevention, whose primary mechanisms involve chelating calcium ions in the lens and inhibiting quinones, thereby reducing lens protein aggregation and opacification [13].

Proteomics, particularly through mass spectrometry (MS), is a powerful approach for investigating DEPs to uncover molecular mechanisms involved in disease processes and therapeutic interventions [14]. In this study, we employed the N-methyl-N-nitrosourea (MNU)-induced cataract model in neonatal Sprague–Dawley (SD) rats. MNU, a DNA alkylating agent, induces cataracts primarily by generating ROS and triggering apoptosis in lens epithelial cells, sharing key pathological features with human age-related cataracts [15]. This model is particularly relevant for testing the antioxidative and anti-apoptotic mechanisms proposed for HRS. This study aimed to explore the protective effects of HRS against MNU-induced cataracts in neonatal Sprague–Dawley (SD) rats. Through proteomic analysis, DEPs were identified between HRS- and normal saline (NS)-treated groups to elucidate the molecular mechanisms underlying the role of HRS in preventing cataracts.

## Materials and methods

### Experimental animals and induction of cataracts

All experimental protocols were approved by the Institutional Animal Care and the Ethics Committee of Academy of Military Medical Sciences (IACUC-2020–3012). The cataract model was established using neonatal SD rats (Crj: CD (SD), Charles River Japan). Rats were balanced by sex and randomly divided into five groups (n = 20 per group): normal control, MNU-only, MNU + NS, MNU + pirenoxine (PRX), and MNU + HRS. MNU (Fluorochem, Hadfield, Derbyshire, UK) was prepared at a concentration of 10 mg/mL by dissolving in 0.05% acetic acid and stored at − 20 °C in a light-protected environment until use. A single intraperitoneal injection of MNU (70 mg/kg body weight) was administered on postnatal day 15 to induce cataracts in all groups except the normal control. HRS was purchased from Beijing Vital Hydrogen Source Biotechnology Co., Ltd. (China). The HRS was prepared by saturating sterile saline with high-purity hydrogen gas under 0.4 MPa pressure, resulting in a dissolved hydrogen concentration consistently maintained above 1.2 ppm, as verified using a hydrogen concentration detector For treatment groups, interventions began on postnatal day 8, consisting of daily intraperitoneal injections of HRS at a dose of 10 mL/kg body weight until day 14, followed by topical eye drops (Santen Pharmaceutical Co., Ltd., Japan) from day 15 to day 21. For the MNU + HRS group, the topical eye drops were HRS (> 1.2 ppm), administered three times daily. PRX, a drug with strong antioxidant properties [16], and NS served as treatment and vehicle controls, respectively.

### Slit-lamp examination and quantification of cataract severity

Slit-lamp examinations were performed to evaluate lens opacities on day 21 following MNU administration. Under anesthesia and after pharmacological mydriasis with 0.5% tropicamide (Santen Pharmaceutical Co., Ltd.), lens opacities were graded using an OPTO-SIL slit-lamp (Optoprobe Science, UK), following the Oxford Clinical Cataract Classification and Grading System [17]. The grading standards were as follows: Grade 0, clear lens; Grade 1, minimal opacity with slight suture marks; Grade 2, moderate opacity; Grade 3, advanced opacity; Grade 4, advanced opacity with visible cracks; Grade 5, severe opacity with radial clouding; and Grade 6, complete opacity with no visible radial patterns. All evaluations were performed by a single investigator following double-blind, randomized procedures to ensure consistency and minimize bias.

### Lens density measurements

Lens density was assessed on day 21 post-MNU administration using the Pentacam anterior segment analyzer (Oculus, Germany). Following 30 min of pharmacological mydriasis induced with compound tropicamide, lens imaging was performed in the enhanced dynamic mode. The scan covered an axis from 90° to 270° with a scanning time of 0.3 s, repeated 15 times to ensure accuracy. Density values were recorded at specific anatomical regions along the corneal vertex axis, including the anterior capsule, subcapsular cortex, anterior cortex (supranuclear region), nucleus (lowest point), nucleus (highest point), posterior cortex, and posterior capsule. Lens density was expressed in grayscale values ranging from 0 (completely transparent) to 100 (completely opaque), and thickness was manually measured. Each measurement was repeated twice, and the mean value was used for analysis.

### Lens transparency measurements

Lens transparency was evaluated using an integrating sphere spectrophotometer (Lambda 950, PerkinElmer, USA) equipped with a customized fixture to secure rat lenses. Baseline calibration was performed for each sample using the fixture, with a reference transmittance of 100% ± 0.5%. Transparency was measured across wavelengths ranging from 360 to 780 nm, with a spectral resolution of 5 nm and sampling intervals of 1 min. Each lens was positioned in the light path, and transmittance data were recorded. Transmittance curves were plotted for each group, and mean transmittance values were calculated for quantitative analysis. Calibration was repeated before measuring each sample to ensure consistency and accuracy.

### Proteomic analysis

Proteomic analysis was performed on lens tissues collected on day 21 post-MNU administration from the MNU + HRS and MNU + NS groups, with six rats per group selected to match sex, age, and body weight. After euthanasia with sodium pentobarbital (100 mg/kg; Beijing Huaye Huayu Chemical Co., Ltd., China), lenses were collected and washed three times with PBS, flash-frozen in liquid nitrogen for 10 min, and stored at -80 °C. Proteins were extracted using lysis buffer (8 M urea, 1% SDS, 3 μM trichostatin A, 50 mM nicotinamide) and protease inhibitors, followed by sonication and centrifugation at 12,000 g for 10 min at 4 °C. Protein concentrations were determined using a BCA assay (Beyotime Biotechnology, Shanghai, China). Extracted proteins were reduced with 5 mM dithiothreitol at 56 °C for 30 min, alkylated with 11 mM iodoacetamide in the dark for 15 min, and diluted to < 2 M urea before trypsin digestion (1:50 enzyme-to-protein ratio) overnight at 37 °C, followed by second digestion (1:100 ratio) for 4 h. Peptides were desalted, dried, and labeled with tandem mass tag (TMT) reagents (Thermo Fisher Scientific, Waltham, MA, USA) according to the manufacturer’s protocol. High-pH reversed-phase HPLC was performed using an Agilent 300Extend C18 column (particle size: 5 μm, dimensions: 4.6 mm × 250 mm; Agilent Technologies, Santa Clara, CA, USA), separating peptides into 60 fractions, which were combined into 18 and vacuum-dried. Peptides were analyzed using an EASY-nLC 1000 system coupled to an Orbitrap Fusion Lumos mass spectrometer (Thermo Fisher Scientific). MS1 scans were recorded at 350–1550 m/z with a resolution of 60,000, and the top 20 precursors underwent higher-energy collisional dissociation (HCD) fragmentation for MS2 scans at 15,000 resolution, using a dynamic exclusion time of 30 s. Raw MS/MS data were searched against the UniProt rat database using MaxQuant (v1.5.2.8), with parameters set for trypsin digestion, up to two missed cleavages, a minimum peptide length of seven amino acids, and 1% false discovery rate for peptide-spectrum match and protein identifications. Quantitative analysis employed TMT-6plex with strict mass tolerances (5 ppm for MS1 and 0.02 Dalton for MS2). Identified proteins were filtered to ensure quantifiability across all groups, with peptide length, mass error, and spectral quality evaluated to ensure data integrity.

### Differential expression analysis

DEPs between the HRS- and NS-treated groups were identified using quantitative proteomic data from three technical replicates per sample. First, the mean protein abundance across replicates was calculated for each sample within each group. Fold changes in protein expression were determined by calculating the ratio of mean values between the two groups. Molecules that were upregulated were defined as those with a t-test *P* value < 0.05 and a fold change (FC) > 1.5. Molecules that were downregulated were defined as those with a t-test *P* value < 0.05 and a fold change (FC) < (1/1.5).Upregulated and downregulated molecules were combined as differentially expressed molecules in each organ.

### Functional enrichment analysis

Enrichment analysis for DEPs was conducted using the Gene Ontology (GO), Kyoto Encyclopedia of Genes and Genomes (KEGG) pathway, and InterPro protein structural domain databases [18]. GO annotation was categorized into three domains: biological processes, cellular components, and molecular functions. KEGG pathway enrichment analysis classified pathways based on hierarchical categorizations. InterPro was used to identify functional protein structural domains. Fisher’s exact two-tailed test was applied to all analyses to assess the enrichment of DEPs relative to the background of all identified proteins. GO terms, KEGG pathways, and protein domains with a *p*-value < 0.05 were considered significantly enriched.

To elucidate potential functional relationships among DEPs, a functional enrichment-based clustering analysis was conducted. DEPs were categorized into four quartiles (Q1 to Q4) based on their fold-change values. For each quartile, enrichment analyses were performed for GO terms, KEGG pathways, and protein structural domains. Functional categories exhibiting significant enrichment (*p*-value < 0.05) in at least one quartile were selected for further analysis. The enrichment *p*-values were transformed to -log10 and subsequently normalized using Z-transformation. Hierarchical clustering was performed employing Euclidean distance and average linkage methods to group related functions across different quartiles. The clustering results were visualized as heatmaps utilizing the heatmap.2 function from the gplots R package. Subcellular localization of DEPs was predicted using the GO database (http://geneontology.org).

### Parallel reaction monitoring (PRM) validation

PRM was conducted to validate TMT-identified DEPs. Targeted peptides were analyzed on a Q Exactive Plus mass spectrometer (Thermo Fisher Scientific). Peptide parameters were set as follows: protease, Trypsin [KR/P]; maximum missed cleavages, 0; peptide length, 7–25 amino acids; fixed modification, carbamidomethylation of cysteine. Transition parameters were: precursor charges 2, 3; product ion charge 1; ion types b, y. Fragment ion selection started from the third to the last ion, with an ion match mass tolerance of 0.02 Da. Peak areas were normalized, and dotp values were calculated to evaluate the confidence of peptide identification. Data acquisition and analysis were performed using Skyline software (v.21.1). Peptide identification confidence was assessed by the dotp value, and only peptides with a dotp value > 0.8 were considered for reliable quantification. Peak areas were normalized against the sample with the highest peptide intensity for relative quantification.

### Statistical analysis

All quantitative data were expressed as mean ± standard deviation. The sample sizes for each experiment were as follows: global functional assessments (slit-lamp, Pentacam, spectrophotometry), n = 20 per group; proteomic analysis, n = 6 per group; PRM validation, n = 3 per group. Statistical analyses were conducted using GraphPad Prism 7 (GraphPad Software, Inc., Boston, MA, USA). Group comparisons were performed using one-way analysis of variance, followed by Duncan's post hoc test to assess intergroup differences. For proteomic and PRM data, proteins with a fold change > 1.5 (or < 1/1.5) and a *p*-value < 0.05 (determined by Student's t-test) were considered statistically significant for differential expression. A *p*-value < 0.05 was considered statistically significant.

## Results

### HRS protects against lens damage in a rat model of mnu-induced cataracts

**Fig. 1:**
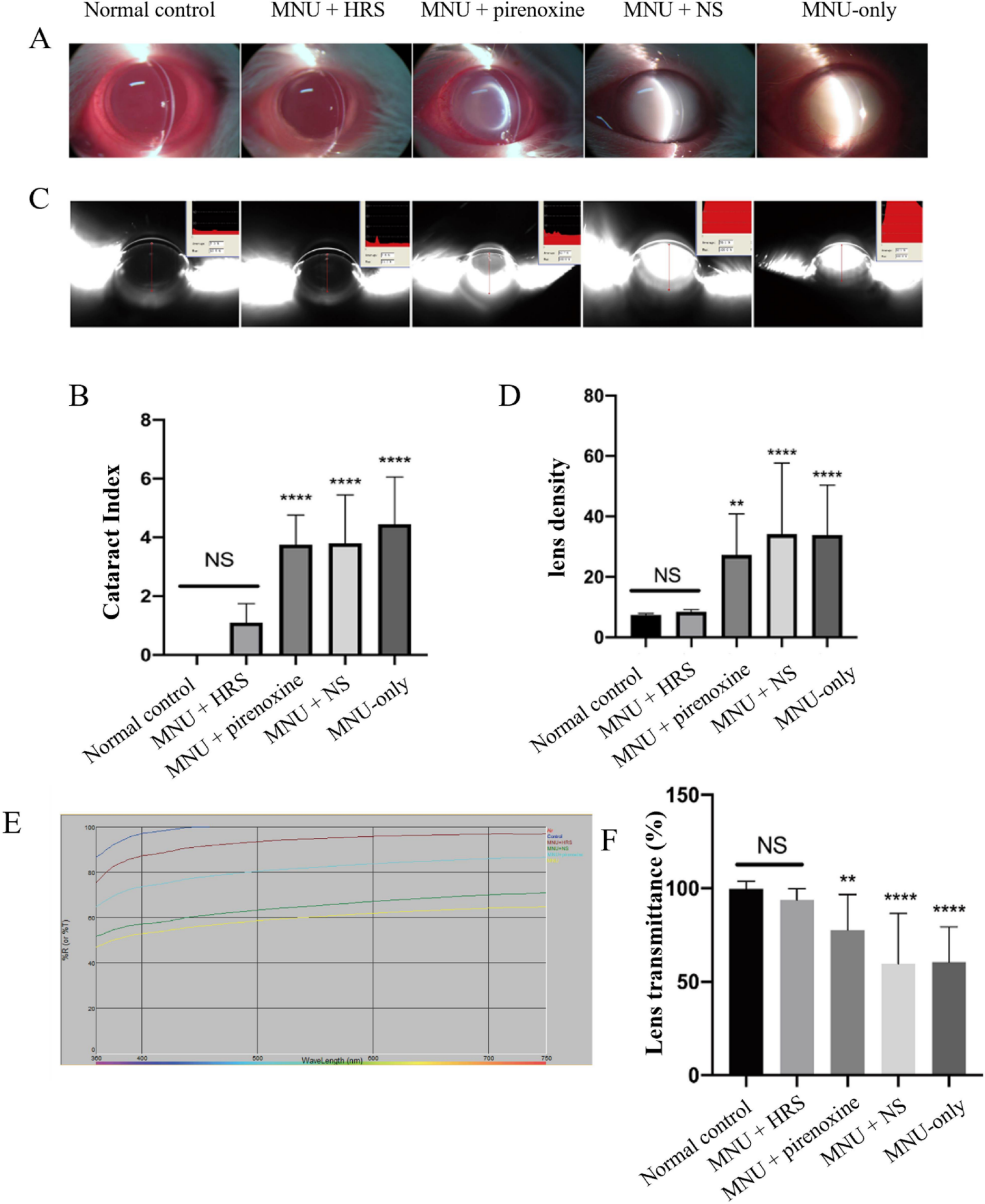
Protective effects of hydrogen-rich saline (HRS) on lens clarity, density, and transparency in N-methyl-N-nitrosourea (MNU)-induced cataracts. **A** Representative slit-lamp images of lens clarity in normal control, MNU-only, MNU + normal saline (NS), MNU + pirenoxine (PRX), and MNU + HRS groups on day 21 post-MNU administration. **B** Quantitative analysis of cataract severity using the cataract index. **C** Representative images of lens density measured by Pentacam in different treatment groups. **D** Quantitative analysis of lens density across groups. **D** Spectral transmittance curves illustrate lens transparency across different groups. **E** Quantitative analysis of lens transparency percentages for each group. Data are presented as mean ± standard deviation (SD). ^**^p < 0.01, ^****^p < 0.0001; ns, non-significatn; n = 10

To evaluate the protective effects of HRS against MNU-induced cataracts, a rat cataract model was established. Slit-lamp examinations on day 21 post-MNU administration revealed no cataract formation in the normal control group, as all lenses remained completely transparent. In contrast, the MNU-only group exhibited severe cataracts with a 100% incidence rate (20/20), predominantly graded at levels 4–5 (18/20). Similar severe lens opacification and a 100% incidence rate were observed in the MNU + NS and MNU + PRX groups. Remarkably, the MNU + HRS group demonstrated a significantly reduced cataract incidence of 50% (10/20), with most opacities graded at levels 1–2 (8/10), indicating mild posterior subcapsular changes (Fig. 1A, Table 1). Cataract severity, quantified by a cataract index, was significantly lower in the MNU + HRS group compared to the MNU-only, MNU + NS, and MNU + PRX groups (*p* < 0.0001; Fig. 1B). These results highlight the protective effect of HRS in reducing both the incidence and severity of MNU-induced cataracts.

**Table 1:** Cataract severity grades across groups

Group	n	Grade 0	Grade 1	Grade 2	Grade 3	Grade 4	Grade 5	Grade 6
Control	20	20 (100%)	0 (0%)	0 (0%)	0 (0%)	0 (0%)		0 (0%)
MNU + HRS	20	10 (50%)	6 (30%)	2 (10%)	2 (10%)	0 (0%)		
MNU + PRX	20		1 (5%)	2 (10%)	6 (30%)	10 (50%)	1 (5%)	0 (0%)
MNU + NS	20			1 (5%)		3 (15%)	11 (55%)	5 (25%)
MNU	20			1 (5%)	1 (5%)	2 (10%)	10 (50%)	6 (30%)

HRS, hydrogen-rich solution; MNU, N-methyl-N-nitrosourea; NS, normal saline; PRX, pirenoxine

### HRS restores lens density and transparency

To further assess the protective role of HRS, lens density and transparency were analyzed. Pentacam anterior segment analysis revealed significantly higher lens density in the MNU-only (34.12 ± 5.280%), MNU + NS (33.90 ± 3.678), and MNU + PRX (27.21 ± 3.057%) groups compared to the normal control group (7.367 ± 0.1437%; all *p* < 0.05). In contrast, the MNU + HRS group had lens density (8.455 ± 0.1570%) comparable to the normal control group (*p* > 0.05) and significantly lower than the MNU-only, MNU + NS, and MNU + PRX groups (all *p* < 0.05; Fig. 1C and D). Lens transparency analysis further supported these findings. Transparency in the MNU-only (59.72 ± 5.868%), MNU + NS (60.62 ± 4.396%), and MNU + PRX (77.63 ± 4.502%) groups was significantly lower than in the normal control group (99.75 ± 0.9212%; all *p* < 0.05). HRS treatment restored lens transparency to 93.86 ± 1.324%, comparable to the normal control group (*p* > 0.05) and significantly higher than the MNU-only, MNU + NS, and MNU + PRX groups (all *p* < 0.05; Fig. 1E and F). These findings confirm that HRS preserves lens clarity and reduces MNU-induced damage.

### High-quality proteomic profiling identifies quantifiable proteins in MNU-induced cataracts

**Fig. 2:**
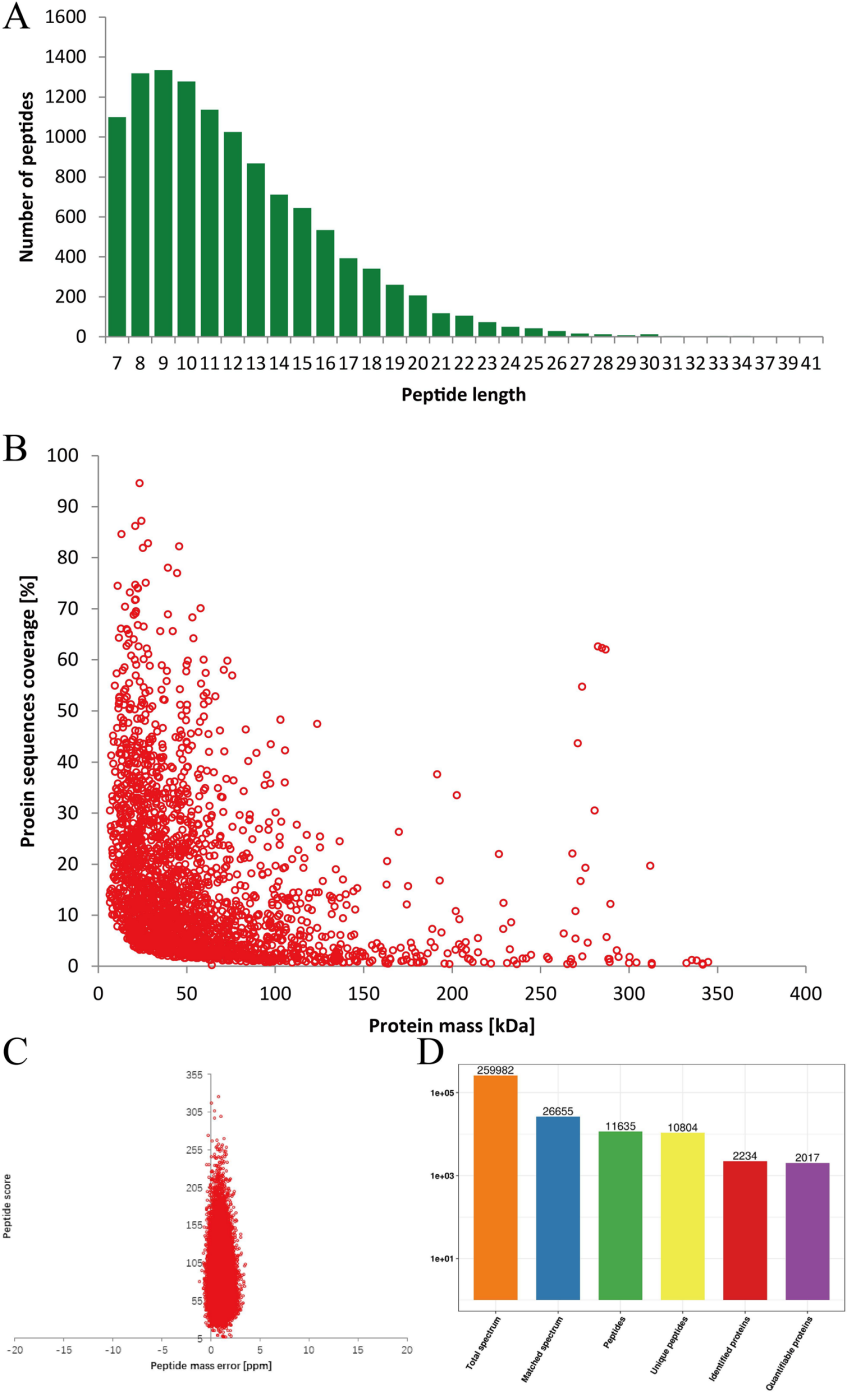
Quality control and summary of proteomic data. **A** Peptide length distribution. **B** Relationship between protein molecular weight and sequence coverage. **C** Peptide mass error distribution. **D** Summary of the proteomic analysis, including the total number of spectra, identified peptides, unique peptides, and quantifiable proteins

To investigate the molecular changes underlying MNU-induced cataracts and the protective effects of HRS, TMT-labeled quantitative proteomics analysis was conducted on lens tissues collected from the MNU + HRS and MNU + NS groups. Rigorous quality control confirmed the reliability of the workflow, with most peptides falling within the optimal length range of 7–20 amino acids for HCD fragmentation, ensuring effective identification (Fig. 2A). Peptide molecular weight analysis revealed a negative correlation between molecular weight and coverage, indicating that larger peptides require more identified fragments to achieve similar coverage compared to smaller peptides (Fig. 2B). Peptide mass error analysis demonstrated high accuracy, with the majority of mass errors falling within ± 5 ppm, a commonly accepted threshold for high-resolution MS, ensuring reliable peptide identification [19]. Peptide scores further confirmed identification confidence, showing a strong inverse correlation with mass error deviations (Fig. 2C). The proteomic analysis generated 259,982 MS/MS spectra, of which 26,655 were deemed effective, yielding a spectral utilization rate of 10.3%. From these spectra, 11,635 peptides were identified, including 10,804 unique peptides, leading to the identification of 2,234 proteins, 2,017 of which were quantifiable (Fig. 2D).

### Identification and characterization of DEPs

**Fig. 3:**
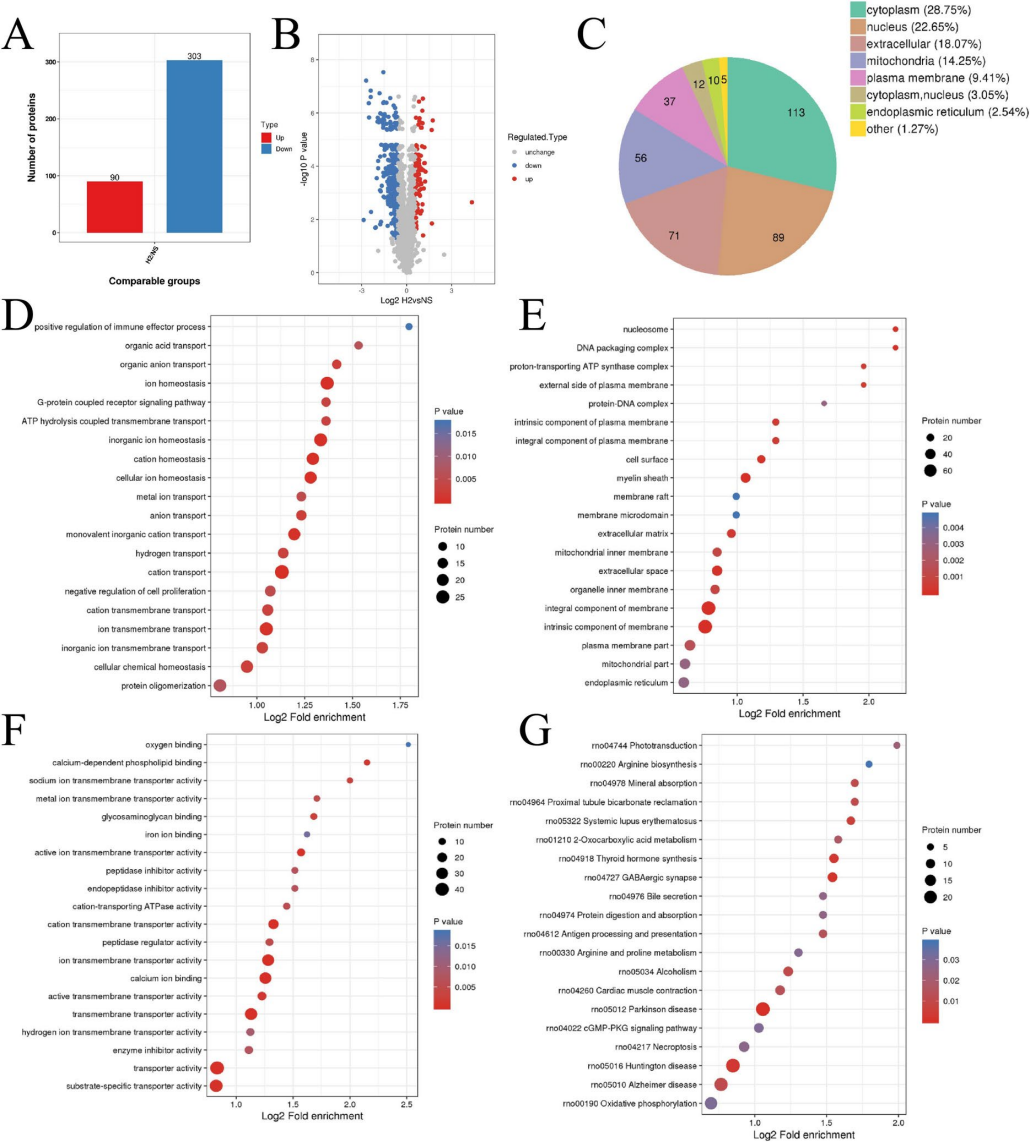
Functional and pathway analysis of differentially expressed proteins (DEPs) in MNU-induced cataracts treated with HRS. **A** The number of DEPs in MNU-induced cataracts treated with HRS compared to those treated with NS. Red indicates upregulated proteins, while blue indicates downregulated proteins. **B** The distribution of significantly upregulated and downregulated proteins. **C** Subcellular localization of DEPs. **D–F** GO enrichment analysis for biological processes **D**, cellular components **E**, and molecular functions **F**. (G) KEGG pathway enrichment analysis. Bubble size represents protein count, color gradient reflects *p*-value significance, with red indicating upregulation and blue indicating downregulation

**Fig. 4:**
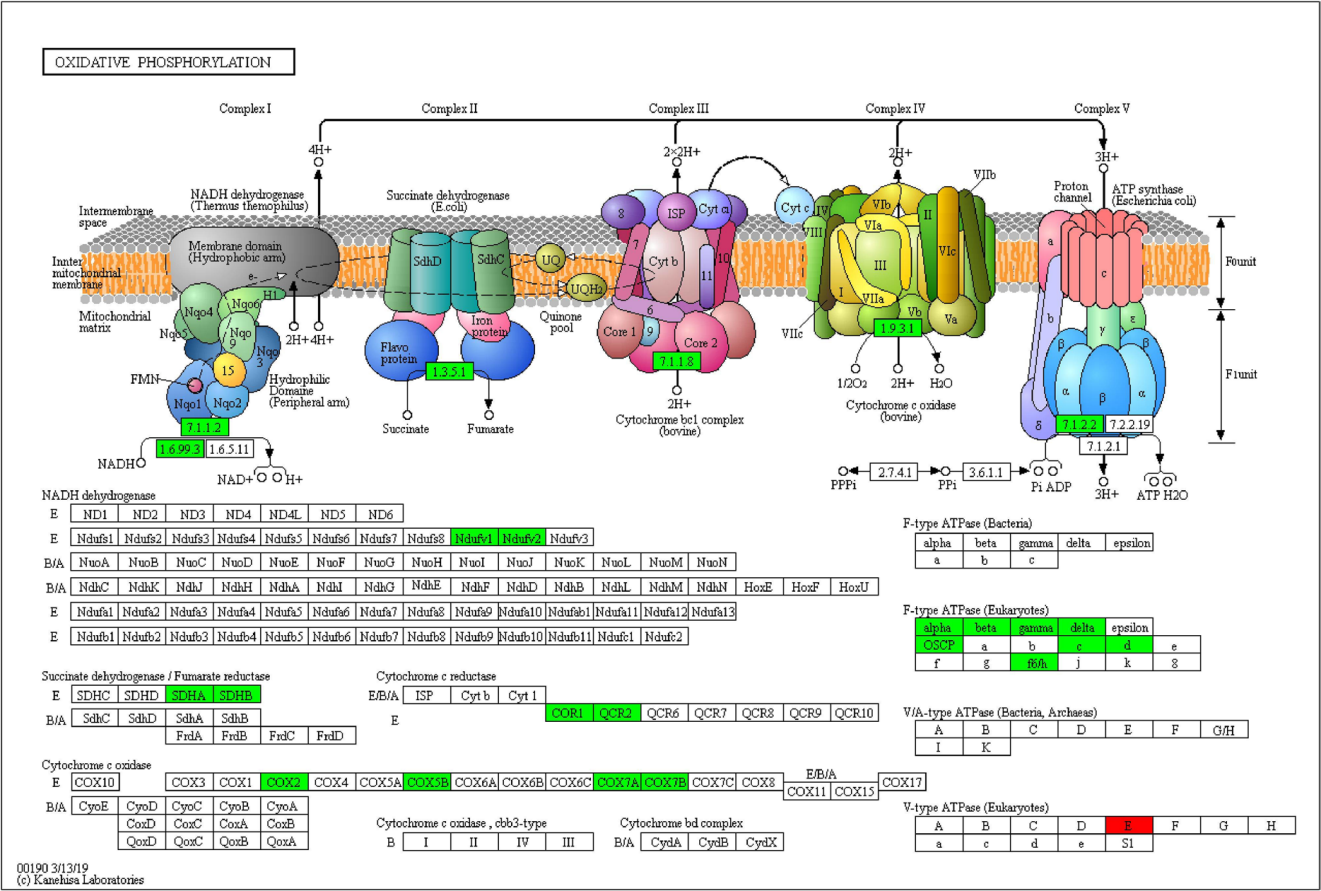
Oxidative phosphorylation pathway showing DEPs in MNU-induced cataracts treated with HRS compared to NS. Proteins in red indicate upregulated expression, while those in green indicate downregulated expression. Nodes in yellow represent multiple proteins with both upregulated and downregulated expression. Key complexes of the electron transport chain (Complexes I–V) are annotated, highlighting significant alterations in mitochondrial metabolic processes associated with cataract progression and the protective effects of HRS treatment

To explore the functional significance of the identified proteins, differential expression analysis and enrichment studies were conducted. Differential expression analysis revealed 90 upregulated and 303 downregulated proteins in the MNU + HRS compared to the NS-treated group (Fig. 3A and B). To ensure data reliability, sample reproducibility was assessed through principal component analysis, which demonstrated distinct clustering of biological replicates (Fig. S1A). Additionally, relative standard deviation analysis indicated low variability (Fig. S1B), and Pearson correlation coefficients showed strong correlations within replicates (Fig. S1C), further validating the consistency and reliability of the proteomics workflow. Subcellular localization analysis revealed that the majority of DEPs were localized to the cytoplasm (28.75%), nucleus (22.87%), and extracellular space (18.07%), with smaller proportions in the mitochondria and plasma membrane (Fig. 3C). GO enrichment analysis revealed that the DEPs are predominantly involved in processes related to ion transport, homeostasis, and transmembrane activity (Fig. 3D). Enriched cellular components included the nucleosome, plasma membrane, and mitochondria (Fig. 3E), while molecular functions such as ion transmembrane transporter activity, peptidase activity, and calcium binding were significantly represented (Fig. 3F). KEGG analysis identified pathways including arginine biosynthesis, mineral absorption, and oxidative phosphorylation (Fig. 3G). To further investigate mitochondrial function, proteins involved in the oxidative phosphorylation pathway were analyzed. Differential expression was observed in key components of mitochondrial complexes I, II, III, IV, and V, suggesting disruptions or adaptations in energy metabolism. These changes were visualized in the oxidative phosphorylation pathway map (Fig. 4), highlighting alterations in proteins critical for electron transport and ATP synthesis. These findings underscore the role of HRS in modulating oxidative stress and restoring mitochondrial function in MNU-induced cataracts.

### Protein clustering and functional enrichment

**Fig. 5:**
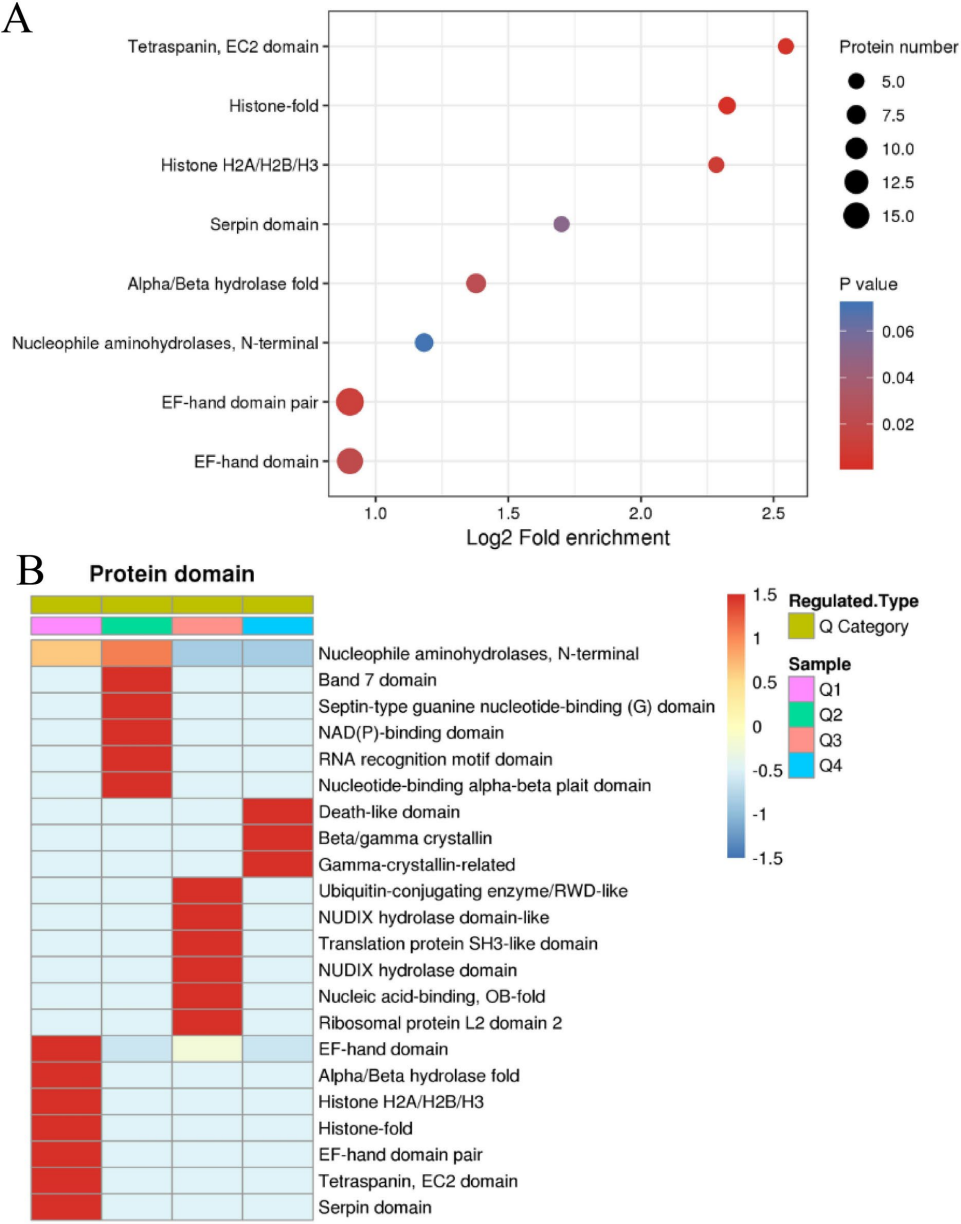
Protein domain enrichment in DEPs. **A** Protein domain enrichment analysis. The bubble size represents the number of proteins, and the color gradient indicates p-value significance. **B** The distribution of enriched protein domains among the Q1–Q4 groups

**Fig. 6:**
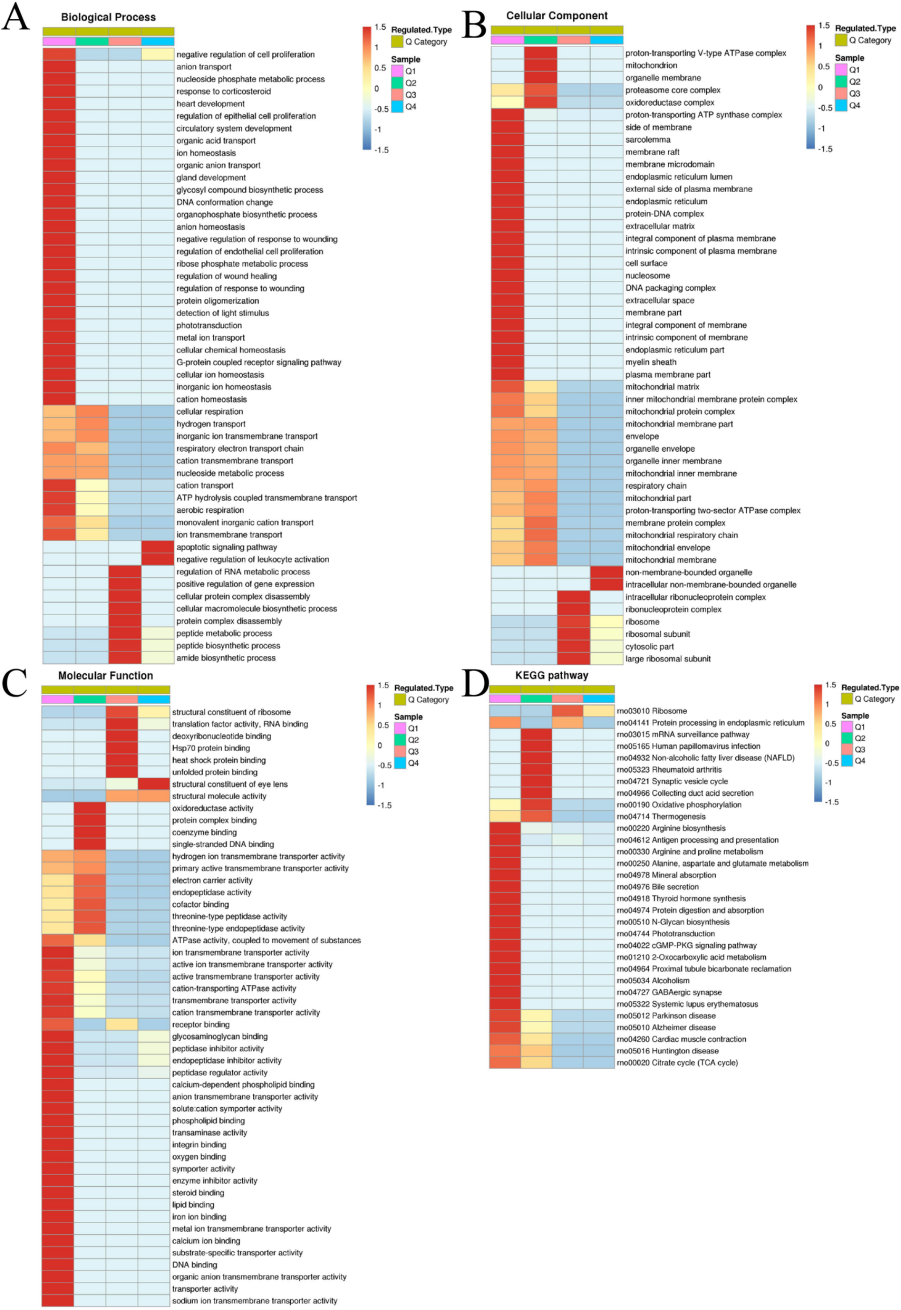
Functional enrichment analysis of Q1–Q4 groups of DEPs in HRS-treated lenses. **A** Biological process enrichment analysis. **B** Cellular component enrichment analysis. **C** Molecular function enrichment analysis. **D** KEGG pathway enrichment analysis

To further investigate the structural and functional features of the DEPs, enrichment and clustering analyses were conducted. Protein domain enrichment analysis identified significant enrichement of domains such as nucleophile aminohydrolases, EF-hand domains, and histone-fold domains, reflecting their critical roles in enzymatic activity, calcium signaling, and chromatin dynamics (Fig. 5A). Heatmap clustering divided proteins into four groups (Q1–Q4) based on fold changes in expression (Fig. 5B), with Q1 exhibiting the strongest enrichment across all GO categories compared to Q2, Q3, and Q4 (Fig. 6). GO biological process analysis revealed that Q1 proteins were significantly enriched in processes regulating cell proliferation, ion and anion transport, metabolic pathways, cellular homeostasis, developmental processes, and responses to external stimuli (Fig. 6A). Cellular component analysis indicated notable enrichment in structural and functional complexes, such as the proton-transporting ATP synthase complex, endoplasmic reticulum (ER), extracellular matrix, plasma membrane, and nucleosome (Fig. 6B). Molecular function enrichment emphasized essential activities including glycosaminoglycan, phospholipid, and DNA binding, as well as calcium-dependent transporter activity and enzyme inhibition (Fig. 6C). KEGG pathway enrichment analysis further identified critical pathways associated with energy and metabolic regulation, including oxidative phosphorylation, ribosome biogenesis, and arginine biosynthesis (Fig. 6D). These findings underscore the involvement of DEPs in key metabolic, structural, and regulatory processes, supporting the protective effects of HRS against MNU-induced cataracts.

### Validation of DEPs confirms protective mechanisms

To validate the TMT findings, PRM analysis was conducted on 15 selected proteins, successfully quantifying 14 of them (Table 2). For example, the peptides GIVIATGDR (corresponding to ATPase Na^+^/K^+^ Transporting Subunit Alpha 2 (ATP1A2), P06686), LAPEYEAAATR (corresponding to protein disulfide isomerase A3 (PDIA3), A0A0H2UHM5), and LLLQVQHASK (corresponding to Adenine Nucleotide Translocator 1 (SLC25A4), Q6P9Y4) exhibited significantly lower normalized peak areas in the HRS-treated group compared to the NS-treated group, consistently across replicates (Fig. S2). Similar downregulation patterns were observed in 11 other proteins, including heat shock protein family A (HSP70) member 5 (HSPA5; P06761), heat shock protein family A (HSP70) member 1B (HSPA1B; P0DMW1), glutamine synthetase (GLUL; P09606) (Fig. S3), protein disulfide-isomerase (P4HB; P04785), voltage-dependent anion-selective channel protein 2 (VDAC2; P81155), heat shock protein 90 beta member 1 (HSP90B1; A0A0A0MY09) (Fig. S4), voltage-dependent anion-selective channel protein 1 (VDAC1; Q9Z2L0), ATP synthase subunit beta (ATP5F1B; G3V6D3), ATP synthase subunit alpha (ATP5F1A; P15999) (Fig. S5), calreticulin (CALR; P18418), and glutamate dehydrogenase 1 (GLUD1; P10860) (Fig. S6). These results suggest that HRS mitigates MNU-induced stress by downregulating stress response and metabolic proteins, supporting its protective effects observed in the study.

**Table 2:** Differential expression of selected proteins identified in TMT analysis and validated by PRM analysis

Protein Accession	Protein Gene	NS/H2 Ratio	NS/H2 P value	NS/H2 Ratio(TMT)
P06686	Atp1a2	3.76	5.18E-05	2.31
A0A0H2UHM5	Pdia3	3.16	8.33E-05	1.74
Q6P9Y4	Slc25a4	3.40	2.77E-04	1.70
P06761	Hspa5	1.24	8.70E-03	2.18
P0DMW1	Hspa1b	3.60	4.39E-05	2.08
P09606	Glul	13.09	3.33E-06	3.02
P04785	P4hb	4.18	1.07E-04	2.12
P81155	Vdac2	3.36	9.19E-04	1.96
A0A0A0MY09	Hsp90b1	2.51	8.17E-05	1.79
Q9Z2L0	Vdac1	4.55	3.86E-05	1.83
G3V6D3	Atp5f1b	4.02	1.02E-04	1.62
P15999	Atp5f1a	3.14	2.48E-05	1.72
P18418	Calr	3.29	5.08E-05	1.98
P10860	Glud1	4.14	2.06E-05	2.13

TMT: tandem mass tag; PRM: parallel reaction monitoring

## Discussion

This study demonstrated that HRS significantly mitigates lens damage in an MNU-induced cataract model. HRS treatment reduced cataract incidence and severity, restored lens density and transparency to levels comparable to normal controls, and modulated protein expression patterns associated with oxidative stress and metabolic dysregulation. Proteomic analysis revealed 393 DEPs in HRS-treated lenses, with enrichment in key pathways such as oxidative phosphorylation and cellular transport. Notably, HRS demonstrated superior protective effects compared to the positive control PRX, as evidenced by a significantly lower cataract incidence and higher lens transparency (Fig. 1, Table 1). This suggests that HRS may act through a broader or more potent mechanism, targeting fundamental processes like energy metabolism and cellular stress responses beyond the calcium chelation and anti-aggregation effects primarily attributed to PRX. These findings highlight the potential clinical significance of HRS as a non-surgical therapeutic strategy to prevent or delay cataract progression by addressing oxidative damage at a molecular level.

HRS has shown promise in treating cataracts and various ocular diseases by utilizing its antioxidative, anti-inflammatory, and anti-apoptotic properties. In cataract models, intraperitoneal injection of HRS increased the activity of antioxidant enzymes such as superoxide dismutase and glutathione reductase, while reducing lipid peroxidation and maintaining lens transparency [20]. For other eye diseases, including corneal endothelial dysfunction and diabetic retinopathy, HRS demonstrated efficacy by reducing oxidative stress, inhibiting apoptosis, and preserving physiological function through mechanisms involving the NF-κB/NLRP3 and FOXO3a/p53/p21 pathways [21]. In retinal ischemia/reperfusion and optic nerve injury models, HRS mitigated oxidative stress and apoptosis through pathways that regulate PARP-1 overactivation [22] and reduce lipid peroxidation [23]. Integrating our results, which demonstrated that HRS protects against oxidative stress-induced cellular damage, aligns with its broader protective effects in ocular models and reaffirms its potential to mitigate damage in various retinal and corneal conditions.

Current proteomics studies on cataracts have focused on identifying the role of protein aggregation, post-translational modifications, and oxidative stress in cataractogenesis. Key findings include the identification of age-related changes in protein solubility, structural modifications such as truncation and cross-linking, and oxidative damage. These processes contribute to the loss of transparency and function in the lens [24]. The novelty of our study lies in elucidating the specific molecular mechanisms by which HRS mitigates cataract formation. Our study demonstrated that HRS influences key components of the oxidative phosphorylation pathway, including mitochondrial complexes I-V. Dysregulation of these components, commonly observed in cataractogenesis, leads to impaired energy metabolism and increased oxidative stress. For example, alterations in complex III stability and associated enzymes can disrupt the proton gradient essential for ATP synthesis, which may be linked to the mechanisms of cataractogenesis [25]. Similarly, mitochondrial dysfunction in cataractogenesis has been associated with enhanced cytochrome c oxidase activity under elevated temperatures, which increases ATP production in lens epithelial cells, driving their proliferation and contributing to nuclear cataract development by increasing lens opacity and altering cellular homeostasis [26]. By modulating these pathways, HRS appears to mitigate the mitochondrial dysfunction associated with cataract formation, contributing to the preservation of lens transparency.

Furthermore, HRS influenced ion transport and metabolite exchange by regulating proteins such as ATP1A2, VDAC1, VDAC2, and SLC25A4. Dysregulation of Na,K-ATPase, including its subunits like Atp1a2, leads to altered Na^+^ and K^+^ levels, increased intracellular calcium, and activation of calcium-dependent proteolytic enzymes, resulting in lens opacity and cataract formation [27]. Additionally, disruption of ion channels such as VDAC1 and VDAC2 contributes to mitochondrial calcium overload and oxidative stress, further exacerbating cataractogenesis by impairing lens transparency and cellular homeostasis [28]. Mutations in *Slc25a4* are associated with mitochondrial dysfunction, causing mtDNA depletion or multiple deletions that disrupt ATP production, impair ionic balance, and compromise cellular homeostasis in the lens, contributing to cataract formation [29]. By modulating these pathways and restoring ionic and metabolic balance, HRS offers a promising therapeutic approach to mitigate cataractogenesis.

HRS may also reduce oxidative stress and protein misfolding by downregulating stress-responsive proteins, such as HSPA5, HSPA1B, HSP90B1, PDIA3, and P4HB, which are involved in protein folding, disulfide bond formation, and cellular stress responses. HSPA5, HSPA1B, and HSP90B1 are stress-responsive chaperones involved in protein folding [30], with HSPA5 and HSP90B1 functioning in the ER [31] and HSPA1B operating in the cytosol to prevent protein aggregation and manage cellular stress [32]. While previous studies have shown that HSP70 and HSP90 are upregulated in response to oxidative stress and play protective roles in maintaining lens transparency [33], the downregulation observed in our HRS-treated model is most parsimoniously interpreted as a consequence of reduced chronic stress signaling, allowing cellular homeostasis to be restored. This view is strongly supported by the concomitant improvement in lens function and the recovery of multiple metabolic pathways. However, an alternative explanation that HRS might directly or indirectly suppress the chaperone system, potentially impairing the lens's ability to cope with acute proteotoxic stress, cannot be entirely ruled out. Given the short-term nature of our model and the lack of direct measurement of ER-stress markers (e.g., CHOP, ATF4) or protein aggregation dynamics, future studies are warranted to definitively distinguish between these possibilities. PDIA3 and P4HB are essential for disulfide bond formation and isomerization in the ER [34, 35], processes that are critical for maintaining the structural stability of lens proteins [36]. Under oxidative stress, abnormal disulfide exchange in proteins like crystallins can promote aggregation and lens opacity [37]. Downregulating PDIA3 and P4HB may reduce aberrant disulfide bond formation and protein aggregation, alleviating the oxidative stress burden and preserving lens transparency. This modulation of ER-associated oxidative pathways highlights the broader protective mechanisms of HRS in cataract prevention.

Mitochondrial ATP synthase subunits ATP5F1B and ATP5F1A are crucial for ATP production, and their downregulation by HRS may reflect a reduction in excessive energy demands caused by oxidative stress in cataractous lenses [8]. Restoring ATP balance through HRS could mitigate energy deficits associated with lens opacity. Similarly, CALR, an ER chaperone that regulates calcium signaling, plays a crucial role in maintaining calcium homeostasis [38], and its downregulation may help mitigate aberrant calcium release and the subsequent activation of proteolytic enzymes, both of which are key contributors to cataractogenesis [39]. In addition, the downregulation of nitrogen metabolism enzymes such as GLUL and GLUD1 by HRS may reduce the accumulation of metabolic byproducts like ammonia, alleviating oxidative and metabolic stress [40]. These findings suggest that HRS exerts its therapeutic effects by targeting dysregulated pathways in energy metabolism, calcium homeostasis, and amino acid metabolism, all of which are critical for lens transparency.

In addition, our enrichment analysis revealed pathways such as arginine biosynthesis and ribosome biogenesis as significantly modulated by HRS. Arginine plays a critical role in maintaining lens transparency by enhancing the chaperone-like activity of α-crystallins, as demonstrated by its ability to increase hydrophobic surface exposure, promote subunit exchange, and delay cataract formation in animal models [41]. In addition, ribosome biogenesis has been implicated in age-related cataract formation. Decreased expression of ribosomal proteins has been associated with lens opacification, suggesting that disruptions in ribosomal function may contribute to cellular dysfunction and protein aggregation in the lens [42]. The enrichment of arginine biosynthesis observed in our study may have implications for lens homeostasis and protection against cataractogenesis.

This study highlights the potential of HRS in mitigating cataractogenesis through its effects on oxidative stress, protein folding, and energy metabolism. While the findings are robust, future investigations could explore the long-term impact of HRS on lens health, investigate further into the underlying molecular pathways, and validate a broader range of proteins identified in the proteomic analysis. These efforts would enhance the understanding of HRS as a therapeutic strategy for cataracts and provide comprehensive insights into its protective mechanisms.

This study has several limitations that should be considered. First, while our proteomic and PRM data robustly identify molecular changes associated with HRS protection and are supported by clear physiological improvements in lens clarity, they primarily establish correlation. Functional validation, such as genetic manipulation (e.g., knockdown or overexpression) of key DEPs (e.g., HSPA5, ATP1A2) in lens epithelial cells or animal models, is required to confirm their causal roles in the observed protective phenotype. Second, the endpoint of this study was 21 days post-MNU administration, which evaluates the early protective effects of HRS but does not address its long-term efficacy or potential chronic toxicity. Future studies with extended follow-up into adulthood, systematic toxicological assessments, and investigation of different dosing regimens are necessary to fully support its clinical translation. Finally, direct measurements of ER-stress markers, protein aggregation dynamics, and temporal changes in apoptotic pathways would provide deeper mechanistic insights, which were beyond the scope of this initial investigation.

In conclusion, this study demonstrates the effectiveness of HRS in mitigating MNU-induced cataracts by reducing lens opacity and restoring transparency. High-quality proteomic profiling revealed that HRS treatment modulates critical molecular pathways, including oxidative phosphorylation, ion transport, and protein folding, through the differential regulation of key proteins. By targeting oxidative stress, energy metabolism, and cellular homeostasis, HRS provides a multifaceted approach to cataract prevention and treatment. These findings highlight the therapeutic potential of HRS and offer valuable insights into its underlying mechanisms, providing a basis for future translational research.

## Supplementary Information

Below is the link to the electronic supplementary material.

Supplementary file1 (JPG 348 KB) Figure S1: Evaluation of reproducibility and consistency in proteomic data. (A) A principal component analysis (PCA) plot shows the clustering of biological replicates for HRS (red) and NS (blue)-treated groups based on protein quantification data. (B) Relative Standard Deviation (RSD) values within replicates for each group evaluate quantification variability. (C) Pearson correlation analysis assesses the association in protein quantification data between replicates within each group. Red indicates strong positive correlations; blue indicates negative correlations.

Supplementary file2 (JPG 614 KB) Figure S2: Normalized parallel reaction monitoring (PRM) peak area of peptides corresponding to ATPase Na+/K+ transporting subunit alpha 2 (P06686), protein disulfide isomerase A3 (A0A0H2UHM5), and adenine nucleotide translocator 1 (Q6P9Y4): Peptides GIVIATGDR and LIIVEGCQR (P06686), LAPEYEAAATR and LNFAVASR (A0A0H2UHM5), and LLLQVQHASK and GNLANVIR (Q6P9Y4) were analyzed for differential expression between HRS- and NS-treated groups. The bar charts illustrate the normalized peak areas of specific peptides from HRS-treated samples (H2_1, H2_2, H2_3) and NS-treated samples (NS_1, NS_2, NS_3). Each bar is divided into colored segments, with each color representing a different fragment ion detected during mass spectrometry (e.g., y7, y6, y5, b2, b3). The height of each bar indicates the abundance of the peptide in that sample. Numbers above the bars are dotp values, reflecting the confidence level of peptide identification.

Supplementary file3 (JPG 661 KB) Figure S3: Normalized PRM peak area of peptides corresponding to heat shock protein family A (Hsp70) Member 5 (P06761), heat shock protein family A (Hsp70) Member 1B (P0DMW1), and glutamine synthetase (P09606): Peptides VEIIANDQGNR and IINEPTAAAIAAYGLDK (P06761), NQVALNPQNTVFDAK and FEELCSDLFR (P0DMW1), and LVFCEVFK and DIVEAHYR (P09606) were analyzed for validation of differential expression in HRS- and NS-treated groups.

Supplementary file4 (JPG 625 KB) Figure S4: Normalized PRM peak area of peptides corresponding to protein disulfide-isomerase (P04785), voltage-dependent anion-selective channel protein 2 (P81155), and heat shock protein HSP 90-beta (A0A0A0MY09): Peptides THILLFLPK and ILEFFGLK (P04785), LTLSALVDGK and GFGFGLVK (P81155), and SILFVPTSAPR and DISTNYYAQSK (A0A0A0MY09) were analyzed for validation of differential expression in HRS- and NS-treated groups.

Supplementary file5 (JPG 625 KB) Figure S5: Normalized PRM peak area of peptides corresponding to voltage-dependent anion-selective channel protein 1 (Q9Z2L0), ATP synthase subunit beta (G3V6D3), and ATP synthase subunit alpha (P15999): Peptides LTFDSSFSPNTGK and LTLSALLDGK (Q9Z2L0), IPVGPETLGR and FTQAGSEVSALLGR (G3V6D3), and APGIIPR and LELAQYR (P15999) were analyzed to validate differential protein expression between HRS- and NS-treated groups.

Supplementary file6 (JPG 777 KB) Figure S6: Normalized PRM peak area of peptides corresponding to calreticulin (P18418) and glutamate dehydrogenase 1 (P10860): Peptides FYGDQEK and GQTLVVQFTVK (P18418), and YSTDVSVDEVK (P10860) were analyzed to validate differential protein expression between HRS- and NS-treated groups.

## Data Availability

All data generated or analyzed during this study are included in this article and supplementary information files.
