# Supplementary figures and images for: Hydrogen-rich saline treatment modulates proteomic profiles to mitigate cataract development in a N-methyl-N-nitrosourea-induced rat model
Source: Int Ophthalmol. 2026 Jan 20;46(1):63. doi: 10.1007/s10792-025-03915-6 (PMC12819484; doi:10.1007/s10792-025-03915-6)

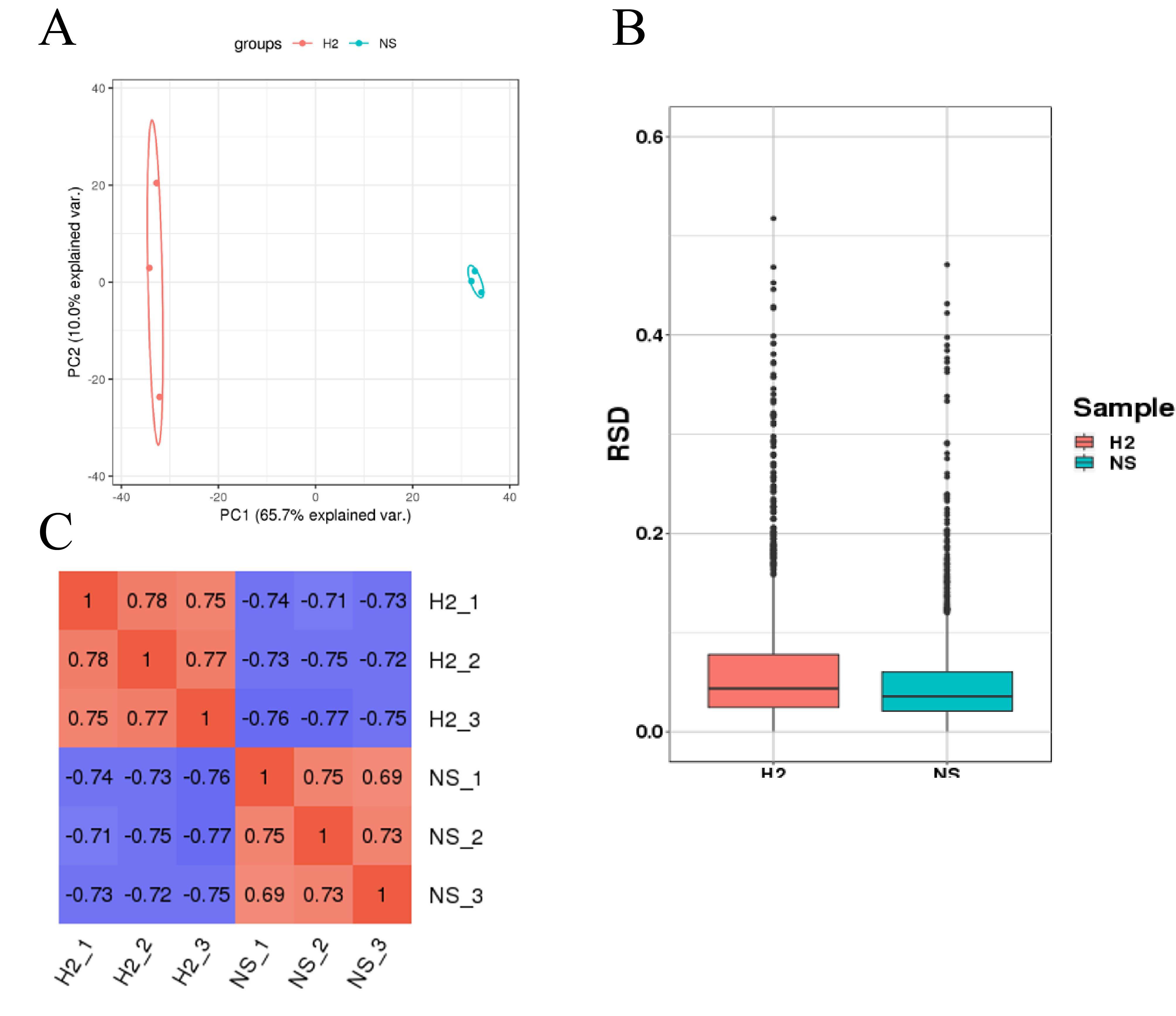

Supplement: Supplementary file 1 — Supplementary file1 (JPG 348 KB) Figure S1: Evaluation of reproducibility and consistency in proteomic data. (A) A principal component analysis (PCA) plot shows the clustering of biological replicates for HRS (red) and NS (blue)-treated groups based on protein quantification data. (B) Relative Standard Deviation (RSD) values within replicates for each group evaluate quantification variability. (C) Pearson correlation analysis assesses the association in protein quantification data between replicates within each group. Red indicates strong positive correlations; blue indicates negative correlations. [file 10792_2025_3915_MOESM1_ESM.jpg]

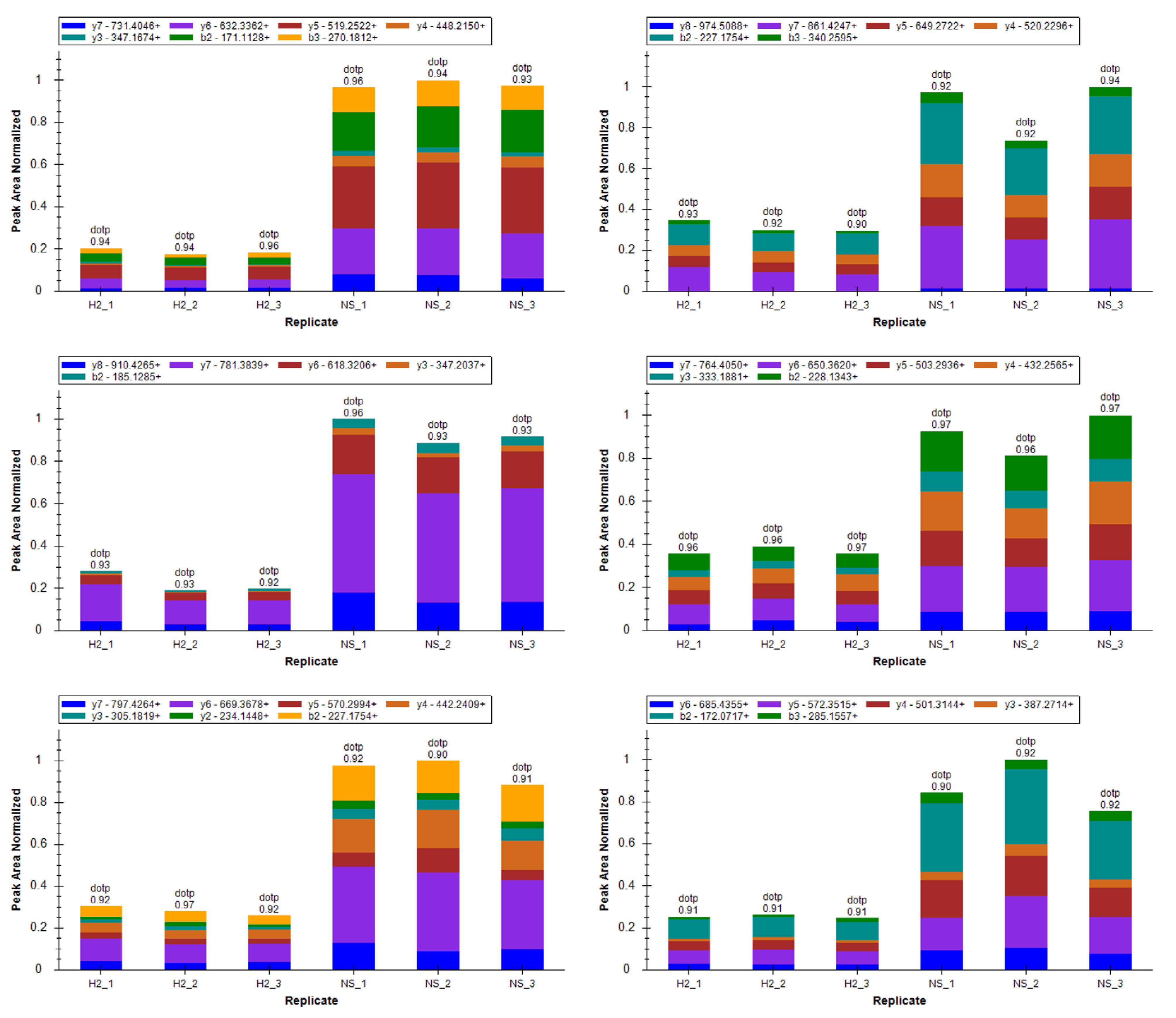

Supplement: Supplementary file 2 — Supplementary file2 (JPG 614 KB) Figure S2: Normalized parallel reaction monitoring (PRM) peak area of peptides corresponding to ATPase Na+/K+ transporting subunit alpha 2 (P06686), protein disulfide isomerase A3 (A0A0H2UHM5), and adenine nucleotide translocator 1 (Q6P9Y4): Peptides GIVIATGDR and LIIVEGCQR (P06686), LAPEYEAAATR and LNFAVASR (A0A0H2UHM5), and LLLQVQHASK and GNLANVIR (Q6P9Y4) were analyzed for differential expression between HRS- and NS-treated groups. The bar charts illustrate the normalized peak areas of specific peptides from HRS-treated samples (H2_1, H2_2, H2_3) and NS-treated samples (NS_1, NS_2, NS_3). Each bar is divided into colored segments, with each color representing a different fragment ion detected during mass spectrometry (e.g., y7, y6, y5, b2, b3). The height of each bar indicates the abundance of the peptide in that sample. Numbers above the bars are dotp values, reflecting the confidence level of peptide identification. [file 10792_2025_3915_MOESM2_ESM.jpg]

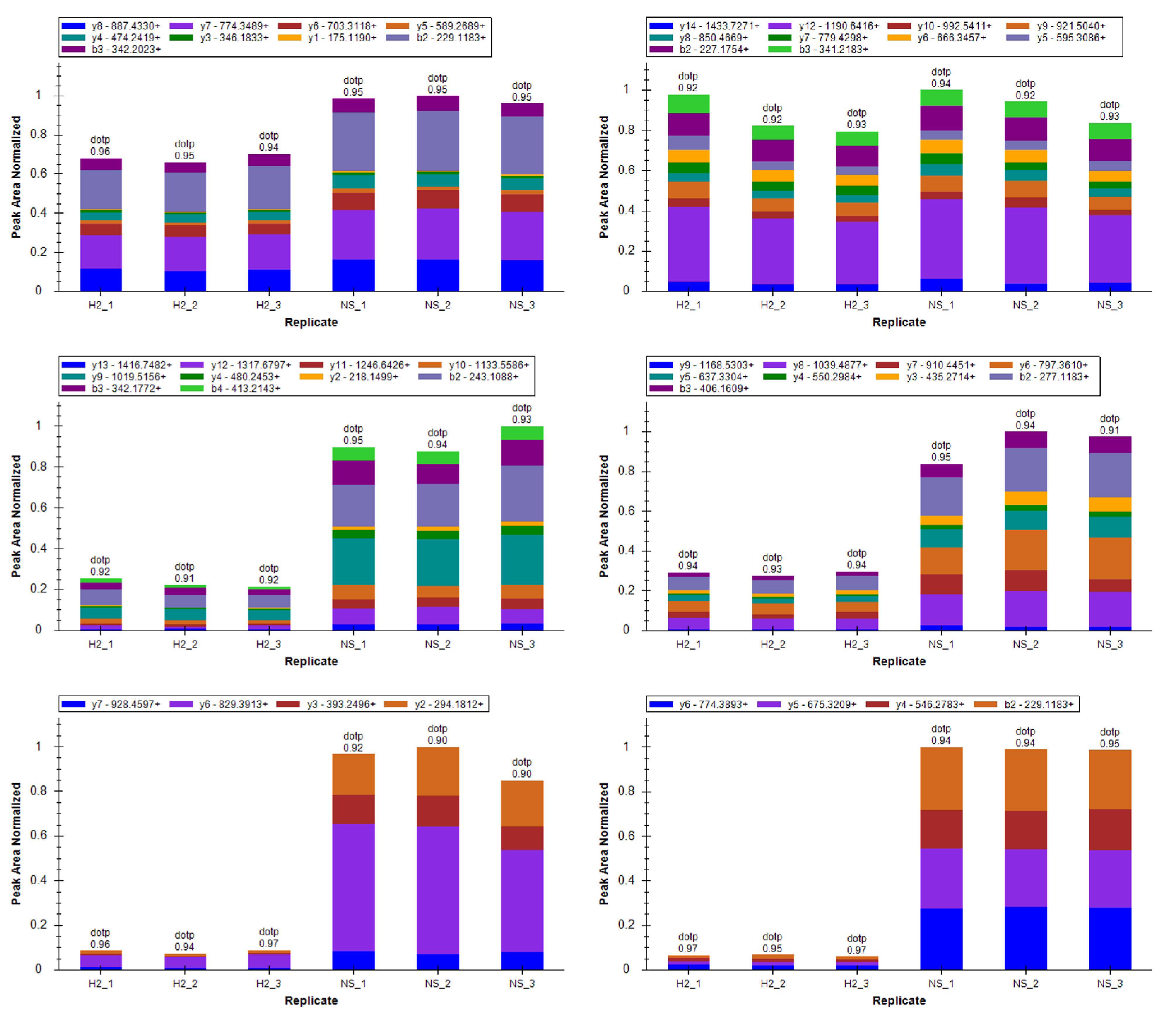

Supplement: Supplementary file 3 — Supplementary file3 (JPG 661 KB) Figure S3: Normalized PRM peak area of peptides corresponding to heat shock protein family A (Hsp70) Member 5 (P06761), heat shock protein family A (Hsp70) Member 1B (P0DMW1), and glutamine synthetase (P09606): Peptides VEIIANDQGNR and IINEPTAAAIAAYGLDK (P06761), NQVALNPQNTVFDAK and FEELCSDLFR (P0DMW1), and LVFCEVFK and DIVEAHYR (P09606) were analyzed for validation of differential expression in HRS- and NS-treated groups. [file 10792_2025_3915_MOESM3_ESM.jpg]

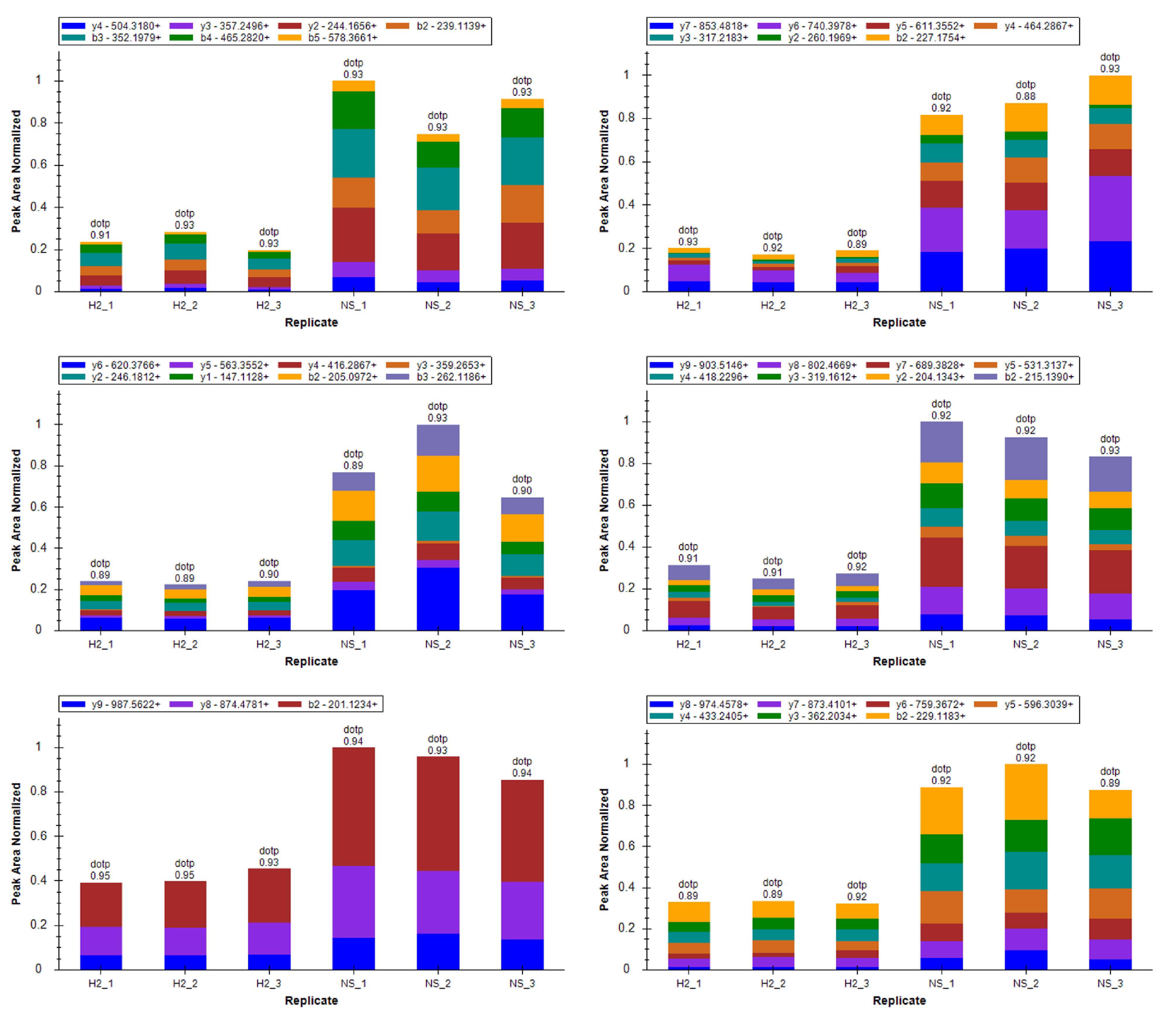

Supplement: Supplementary file 4 — Supplementary file4 (JPG 625 KB) Figure S4: Normalized PRM peak area of peptides corresponding to protein disulfide-isomerase (P04785), voltage-dependent anion-selective channel protein 2 (P81155), and heat shock protein HSP 90-beta (A0A0A0MY09): Peptides THILLFLPK and ILEFFGLK (P04785), LTLSALVDGK and GFGFGLVK (P81155), and SILFVPTSAPR and DISTNYYAQSK (A0A0A0MY09) were analyzed for validation of differential expression in HRS- and NS-treated groups. [file 10792_2025_3915_MOESM4_ESM.jpg]

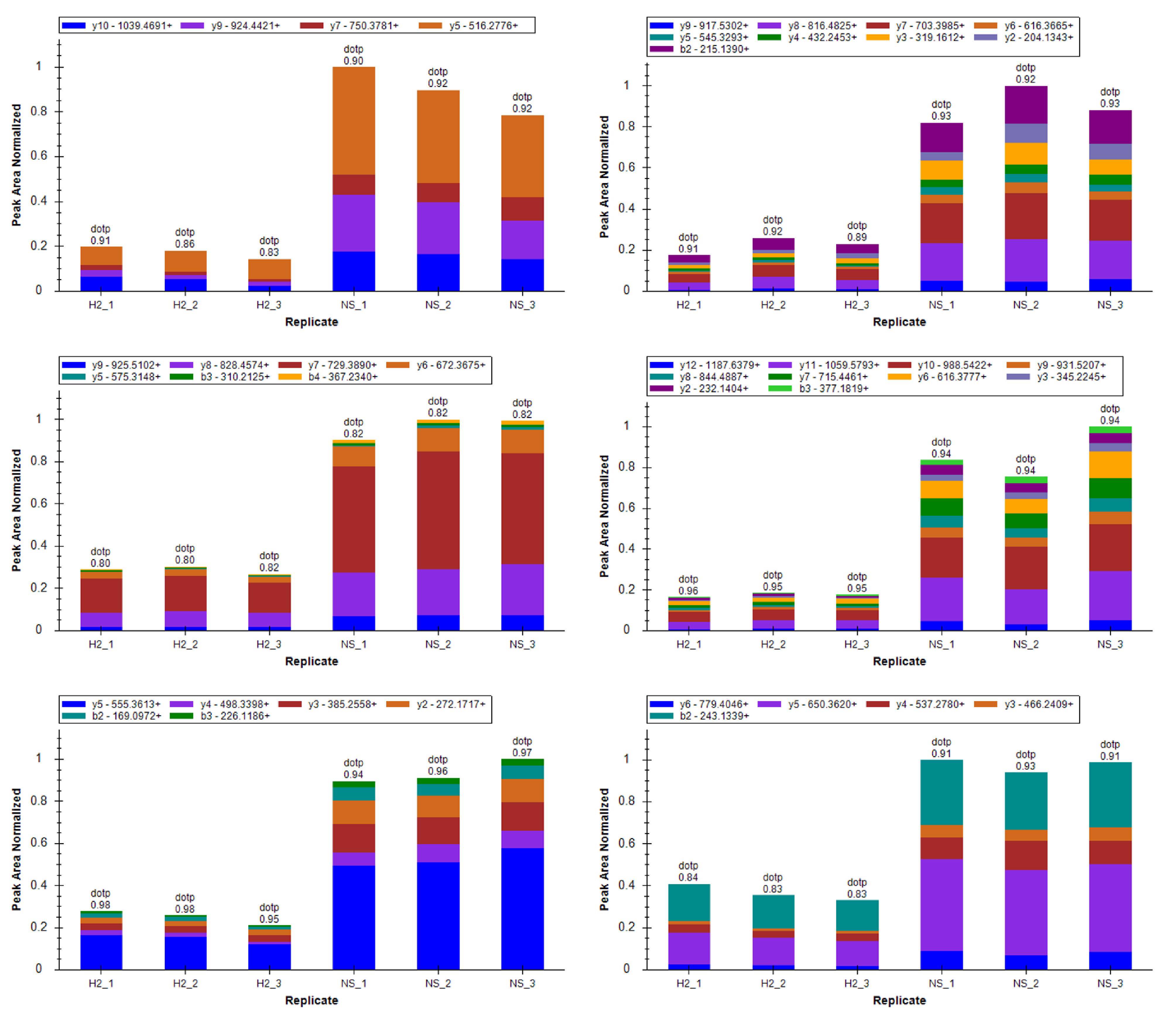

Supplement: Supplementary file 5 — Supplementary file5 (JPG 625 KB) Figure S5: Normalized PRM peak area of peptides corresponding to voltage-dependent anion-selective channel protein 1 (Q9Z2L0), ATP synthase subunit beta (G3V6D3), and ATP synthase subunit alpha (P15999): Peptides LTFDSSFSPNTGK and LTLSALLDGK (Q9Z2L0), IPVGPETLGR and FTQAGSEVSALLGR (G3V6D3), and APGIIPR and LELAQYR (P15999) were analyzed to validate differential protein expression between HRS- and NS-treated groups. [file 10792_2025_3915_MOESM5_ESM.jpg]

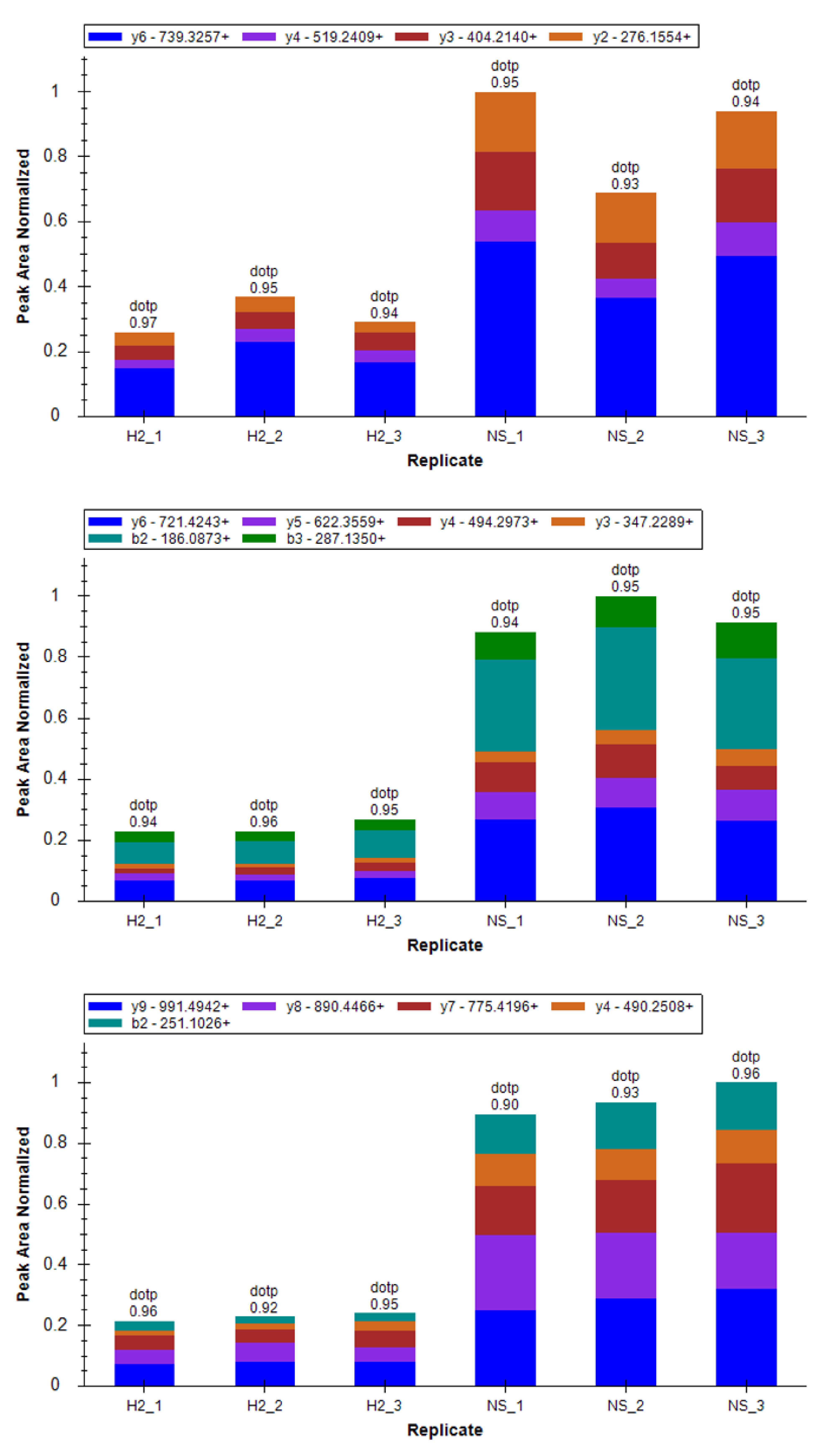

Supplement: Supplementary file 6 — Supplementary file6 (JPG 777 KB) Figure S6: Normalized PRM peak area of peptides corresponding to calreticulin (P18418) and glutamate dehydrogenase 1 (P10860): Peptides FYGDQEK and GQTLVVQFTVK (P18418), and YSTDVSVDEVK (P10860) were analyzed to validate differential protein expression between HRS- and NS-treated groups. [file 10792_2025_3915_MOESM6_ESM.jpg]
